# A simple test‐based frailty index to predict survival among cancer patients with an unplanned hospitalization: An observational cohort study

**DOI:** 10.1002/cam4.4107

**Published:** 2021-08-05

**Authors:** Timothy Hembree, Olga Theou, Sarah Thirlwell, Richard R. Reich, Biwei Cao, Marina Sehovic, Misbahuddin Syed, Neha Verma, Thu‐Cuc Nguyen, Dinesh Keerty, Jaqueline Wesolow, Viktoriya Koverzhenko, Martine Extermann, Jessica Huang, Asha Ramsakal

**Affiliations:** ^1^ Department of Internal and Hospital Medicine H. Lee Moffitt Cancer Center and Research Institute Tampa FL USA; ^2^ School of Physiotherapy Dalhousie University Halifax Nova Scotia Canada; ^3^ Geriatric Medicine Research Unit Nova Scotia Health Authority Halifax Nova Scotia Canada; ^4^ Department of Supportive Care Medicine H. Lee Moffitt Cancer Center and Research Institute Tampa FL USA; ^5^ Biostatistics Core H. Lee Moffitt Cancer Center and Research Institute Tampa FL USA; ^6^ Senior Adult Oncology Program H. Lee Moffitt Cancer Center and Research Institute Tampa FL USA; ^7^ University of South Florida Morsani College of Medicine Tampa FL USA

**Keywords:** frailty, hospital mortality, hospitalization, inpatients, patient readmission

## Abstract

**Background:**

Frailty is a state of increased vulnerability to stressors, and predicts risk of adverse outcomes, such as mortality. Frailty can be defined by a frailty index (FI) using an accumulation of deficits approach. An FI comprised of 20 items derived from our previously studied test‐based frailty index (TBFI) and an additional 33 survey‐based elements sourced from the standard CGA was developed to evaluate if predictive validity of survival was improved.

**Methods:**

One hundred eighty‐nine cancer patients during acute hospitalization were consented between September 2018 and May 2019. Frailty scores were calculated, and patients were categorized into four groups: non‐frail (0–0.2), mildly frail (0.2–0.3), moderately frail (0.3–0.4), and severely frail (>0.4). Patients were followed for 1‐year to assess FI and TBFI prediction of survival. Area under the curve (AUC) statistics from ROC analyses were compared for the FI versus TBFI.

**Results:**

Increasing frailty was similarly associated with increased risk of mortality (HR, 4.5 [95% CI, 2.519–8.075] and HR, 4.1 [95%CI, 1.692–9.942]) and the likelihood of death at 6 months was about 11‐fold (odds ratio, 10.9 [95% CI, 3.97–33.24]) and 9.73‐fold (95% CI, 2.85–38.50) higher for severely frail patients compared to non‐frail patients for FI and TBFI, respectively. This association was independent of age and type of cancer. The FI and TBFI were predictive of survival for older and younger cancer patients with no significant differences between models in discriminating survival (FI AUC, 0.747 [95% CI, 0.6772–0.8157] and TBFI AUC, 0.724 [95% CI, 0.6513–0.7957]).

**Conclusions:**

The TBFI was predictive of survival, and the addition of an in‐person assessment (FI) did not greatly improve predictive validity. Increasing frailty, as measured by a TBFI, resulted in a meaningfully increased risk of mortality and may be well‐suited for screening of hospitalized cancer patients.

## Graphical abstract

A simplified test based frailty index (TBFI) is similarly predictive of survival as a more comprehensive CGA based frailty index in the hospitalized cancer patient. This index may be useful as a screening tool in the acute cancer patient.

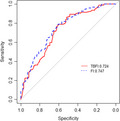

## 1 INTRODUCTION

Cancer prognostication at the time of diagnosis typically is based on factors such as tumor characteristics (e.g., grade and stage), size, and location and depends on the nature and quality of treatment received. Although these factors are useful for estimating survival during the initial phase after diagnosis, they tend to be less helpful for predicting outcomes during later phases of cancer.^1^ For patients with advanced cancer, estimations of survival are more often based on subjective and objective clinical findings regarding a patient's overall status.^2^ Indeed, survival during advanced stages of illness varies based on a multitude of contributing factors. As a state variable, frailty is a means of conceptualizing these cumulative changes resulting in a decline in overall status and poor outcomes.^3^

Frailty is defined as diminished physiologic reserve, resulting in increased vulnerability to adverse outcomes compared with people of the same age.^4^ Although frailty is typically associated with older age, it is important to understand that younger patients outside the geriatric population can be frail as well. This is particularly true for patients with cancer, for whom the disease itself, not age, may be responsible for the most significant decline in physiologic reserve. Recent studies have confirmed that biological/ physiological age is a better predictor of outcomes than chronologic age, and reliance on chronologic age alone can result in the over‐ or undertreatment of patients with cancer.^5^, ^6^, ^7^, ^8^ Frailty is strongly associated with an increased risk of death and worsening health status among patients ≥65 years of age with an acute medical illness.^9^

Diverse medical fields have begun to use frailty in the acute care setting, with in‐hospital disciplines of geriatrics, emergency departments, general medicine, cardiology, and orthopedics most frequently using this metric.^9^ However, frailty is still underexplored in the acute oncology care setting. From 2000 to 2015, there were only six publications that used a frailty assessment for hospitalized patients with cancer.^7^ In these studies, frailty was used to determine limited aspects of patient management. Specifically, frailty was used to identify patients who would benefit from geriatric intervention in the inpatient setting,^10^ monitor referral patterns to palliative services,^11^ determine use of modified chemotherapy regimens,^12^ test feasibility of performing a geriatric assessment of a hospitalized patient,^13^ predict clinical responses and chemotherapy toxicity,^14^ and test for risk of rehospitalization and death.^5^ In an observational study of patients with lung cancer, the prognostic Geriatric 8 tool demonstrated that frail patients with cancer have a significantly greater risk of 1‐year mortality.^15^

The two predominant methods used in oncology for measuring frailty are the Fried Phenotype^16^ and Rockwood Accumulation of Deficits Model frailty index (FI),^17^ and there are numerous iterations of these methods.^3^ The Fried Phenotype has proven difficult to execute for geriatric patients in the inpatient setting because of patients’ cognitive and physical impairments,^18^ whereas the Accumulation of Deficits Model has been shown to be feasible to execute among the hospitalized patient population.^19^

The Comprehensive Geriatric Assessment (CGA) has been considered the gold standard for evaluating frail geriatric patients.^20^ Multiple instruments have been developed and used for calculation of an FI from elements of a CGA and were found to be robust predictors of poor outcomes,^21^ and others have previously used elements of the CGA to calculate an FI for cancer patients.^22^, ^23^ An FI can be constructed using existing clinical‐ and population‐based data, and not every FI needs to include the same items to achieve closely comparable estimates of risk; workable FIs have previously been constructed from several iterations of the standard CGA.^22^ Since 2015, there has been an increase in comprehensive geriatric assessments of hospitalized cancer patients, demonstrating a high prevalence of geriatric conditions.^24^, ^25^, ^26^ Cohen et al demonstrated the conversion of a comprehensive geriatric assessment into an FI and showed that frailty was associated with grade three toxicities during chemotherapy, drug discontinuation, and hospitalization.^27^ An increased understanding of a patient's frailty at the time of an unplanned hospitalization, risk for further decompensation, and prognosis could equip clinicians to make better treatment decisions in alignment with patient needs, values, and wishes for care. This understanding is important, considering the recent studies suggesting that hospitalization and aggressive end‐of‐life (EOL) care for patients with cancer are increasing^28^ despite evidence of associations of aggressive EOL care with poor survival and quality of life (QOL) outcomes.^29^ Rates of in‐hospital deaths, intensive care unit admissions, and unplanned 30‐day readmissions among patients with cancer are alarmingly high. In a cohort of 211 816 American patients with cancer who were aged 65 or older, 22% died in the hospital,^30^ which is of great concern when considering surveys that indicate over 80% of Americans want to die at home.^31^

Identifying those patients for whom survival is limited (i.e., severely frail patients) will allow us to achieve two desired changes in the care of our patients: (1) align medical interventions with patients’ goals regarding QOL and wishes for EOL care and (2) decrease healthcare expenditures for EOL care. Identifying patients who are considered moderately frail would allow for (1) referral for further assessment to identify modifiable risk factors and (2) initiation of interventions to return the patient to a more robust state, which will have a positive impact on cancer outcomes.

We decided to test if adding elements of the CGA to a previously studied TBFI enhances the screening capability of our model. The objectives of this study were to compare a simple TBFI to a more comprehensive FI for prediction of survival among patients with cancer who had an unplanned hospitalization, determine whether biological/physiological age (as measured by frailty) would predict these outcomes independent of chronological age, and illustrate that frailty could be useful in screening both younger and older cancer patients for risk of poor outcomes.

## 2 METHODS

In this observational cohort study, potentially eligible patients were identified through a daily review of hospital admissions. Providers who are part of the project team or a clinical trial coordinator performed an initial assessment of eligibility, and the patient consented to the study. For each patient who consented, eligibility was confirmed later through a more thorough review of the medical record. Data were collected in a prospective manner via the Internal Hospital Medicine (IHM) inpatient medical ward or in the urgent care center at Moffitt Cancer Center (for patients who are admitted to the hospital but resided in the urgent care center). Patients were eligible for the study if they were admitted to the IHM team at Moffitt Cancer Center for an unplanned admission, had English listed as their primary language on their medical record, were able to provide consent, and had a diagnosis of cancer.

From 25 September 2018, until 16 May 2019, 222 patients were approached for study participation, and 206 patients signed consent forms. Reasons for refusal included being too busy, too sick, or not interested (*N* = 7) or no reason was given (*N* = 9). An additional six consented patients were excluded during the second review of eligibility for the following reasons: the patient was Spanish‐speaking (*N* = 2; as a note, both patients were bilingual and proficient in English but had Spanish listed as their primary language in their medical record), their admission was planned (*N* = 1), they had no cancer diagnosis (*N* = 1), and a legally authorized representative signed the consent form without prior IRB approval to use legally authorized representatives for consenting (*N* = 2). Patients with missing lab values were excluded from the analysis (*N* = 3). Patients enrolled in the study who died during the index admission were also excluded from the analysis (*N* = 8). In total, 189 patients were included in the analysis (Figure S1).

Interviews were conducted in a private hospital room. After a patient agreed to participate and signed the study consent form, the patient was interviewed by a study team member. Only family members invited by the patient were present during the interview. One‐time questionnaires and self‐report surveys were administered to patients by the study team member. Additional study data were collected from a review of the patient's electronic health record.

### 2.1 Frailty tools

The FI was constructed in accordance with accepted standards^32^ and followed a deficit accumulation approach. The FI included 53 health‐related items, 20 of which represented the domains of clinical/laboratory tests, healthcare use, and objective cancer‐specific items (such as presence or absence of metastatic disease) and were derived from a previously published cancer‐specific TBFI (Table S1) called the deficit‐accumulation survival index (DASI).^33^ The remaining 33 items represented domains of activities of daily living (ADLs), instrumental ADLs, mobility and fall risks, cognition and memory, Eastern Cooperative Oncology Group (ECOG) performance status, comorbidities, and symptom management, and these items were collected through a self‐reporting survey/questionnaire derived from elements of the comprehensive geriatric assessment (CGA) tool (Table S2). To calculate frailty scores, we divided the summed deficits by the total number of items measured, with the potential scores ranging from zero to one. Higher values represented greater frailty. Patients with missing data were excluded from analysis. All binary variables were recoded using the convention that zero indicated the absence of a deficit and one the presence of a deficit. For variables that included intermediate response(s) (e.g., sometimes or maybe), we used additional values of 0.75, 0.5, and 0.25. Cutoff points were used for continuous variables (as follows).

For laboratory tests, we collected unidentified patient data from 1000 patients seen by the Moffitt Cancer Center IHM team over the same time period as the study and calculated the mean and standard deviation. The cutoff point was set at ±1 SD from the mean. We assigned a zero for scores above the cutoff point and a one for scores below the cutoff point (e.g., albumin >2.5 would equate to zero deficit and albumin ≤2.5 would equate to one deficit). FI scores were categorized into four groups: non‐frail (0–0.2), mildly frail (0.2–0.3), moderately frail (0.3–0.4), and severely frail (>0.4).^34^

### 2.2 Mortality

The outcome variable was all‐cause mortality after the frailty assessment, represented as overall survival (OS). OS was calculated from the date of discharge from the index admission, during which a frailty assessment was performed, to the date of death or censoring. Vital status updates were performed by reviewing the patients’ medical records at the end of each month and at the end of the study (31 January 2020). Time to death was recorded in days. Patients were followed up from the date of index admission until death or until the end of the study follow‐up period. The follow‐up period for all patients was 1 to 472 days (average, 184 days). One hundred and five patients died within 1 to 396 days (average, 82 days) from the date of discharge from index admission.

### 2.3 Statistical analyses

In our cohort (*n* = 189) patient characteristics were compared between frailty categories using a chi‐square test for categorical variables and analysis of variance for continuous variables. OS was tested using a Cox proportional hazards model with the FI as the primary independent variable. To test whether frailty prediction (FI and TBFI) was independent of other factors, the patient's cancer type, age, and sex were added as covariates into this model. Kaplan–Meier curves were used to graphically depict survival outcomes. Statistical significance was defined as *p* ≤ 0.05.

Receiver operating characteristic (ROC) curves were calculated to estimate the areas under the curves (AUCs) for the TBFI and FI in relation to the outcome parameter, mortality at 180 days. Comparisons among the AUCs were performed using the method of DeLong.^35^ Results are given as mean ± standard deviation, median (interquartile ranges), percentage, or AUC (95% confidence intervals) in the text and/or tables. The level of statistical significance was set a priori at *p* ≤ 0.05.

### 2.4 Ethics approval

Individuals whose data are included in this study database have provided written informed consent to allow their data to be recorded and used for research purposes. The study protocol and consent form were approved by the Institutional Review Board of the University of South Florida. All participants provided informed written consent prior to enrollment in the study.

## 3 RESULTS

This study included 189 hospitalized patients treated through the hospitalist service at Moffitt Cancer Center. The mean age ± SD for our cohort was 61.6 ± 12.0 years (range, 26–87 years) and 51.3% of patients were female. Most of our patients identified as White (86.8%), and 8.5% of patients identified as African American/African/Black/Caribbean, 1.6% as Asian/Pacific Islander, 0.5% as Native American/American Indian, and 2.7% as a different race (i.e., selected “Other”). The most common cancer types in our cohort were gastrointestinal (28.6%), lung (20.6%), genitourinary (14.3%), and breast cancers (13.2%). Metastatic disease was seen among 75.1% of patients in our cohort. Twenty percent (*n* = 38/189) and 25% (*n* = 49/189) of our patients were severely frail (score >0.40) as measured by the FI and TBFI, respectively (Table 1). General characteristics of the severely frail group compared to the overall cohort can be reviewed in Table S3 and S4.

**TABLE 1:** Characteristics of the patients included in the study

Characteristic	Patients in study cohort (N=189*)*
Age, year, median (range)	62.0 (26.0;87.0)
<62 years	90 (47.6%)
≥62 years	99 (52.4%)
Sex, No. (%)	
Female	97 (51.3%)
Male	92 (48.7%)
Type of primary cancer, No. (%)	
Breast	25 (13.2%)
Gastrointestinal	54 (28.6%)
Genitourinary	27 (14.3%)
Head and Neck	10 (5.29%)
Lung	39 (20.6%)
Melanoma	11 (5.82%)
Other	23 (12.2%)
Race, No. (%)	
African American/African/Black/Caribbean	16 (8.47%)
Asian/Pacific Islander	3 (1.59%)
Native American/American Indian	1 (0.53%)
Other	5 (2.65%)
White	164 (86.8%)
Metastasis, No. (%)	
0	47 (24.9%)
1 or more sites	142 (75.1%)
FI Frailty Status, No. (%)	
Non‐frail (0–0.2)	59 (31.2%)
Mildly Frail (0.2–0.3)	58 (30.6%)
Moderately Frail (0.3–0.4)	34 (17.9%)
Severely Frail (>0.4)	38 (20.1%)
TBFI Frailty Status, No. (%)	
Non‐frail (0–0.2)	20 (10.5%)
Mildly Frail (0.2–0.3)	62 (32.8%)
Moderately Frail (0.3–0.4)	58 (30.7%)
Severely Frail (>0.4)	49 (25.9%)

Abbreviations: FI, frailty index; TBFI, test‐based frailty index.

In our cohort, 5.8% (*n *= 11/189) of patients were discharged to hospice during the index admission. An additional 17.4% (n = 31/178) of the cohort were discharged to hospice during a subsequent admission. There were 51 total hospitalizations between these 31 patients. Ultimately, among the patients who were discharged to hospice, the median length of stay for hospice admissions was only 12 days.

### 3.1 Frailty indices (FI and TBFI)

**FIGURE 1:**
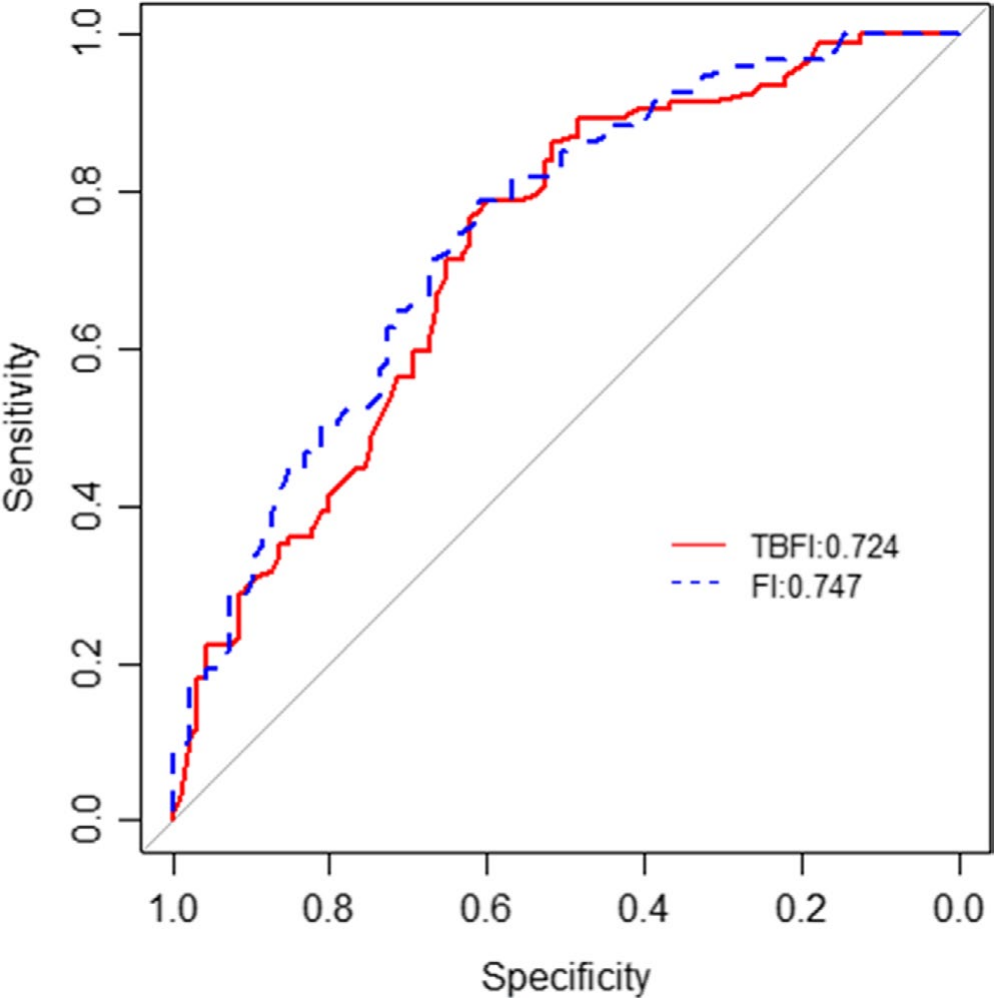
AUC curves for TBFI and FI model

The FI and TBFI had the general characteristics of a normal distribution, with mean ± SD score (on a 0–1 scale) of 0.28 ± 0.12 and 0.35 ±  0.11, respectively. The 99% limit to deficit accumulation was below the theoretical maximum of 1.0, at 0.56 and 0.60 for FI and TBFI, respectively. Chronological age was not associated with FI or TBFI scores (Figures S2 and S3), and the model with FI had a slightly larger AUC compared to the TBFI. However, DeLong's test for the two ROC curves showed that the difference between the AUCs was not significant (*p* = 0.49). The 95% CI of AUC was 0.65 to 0.80 for the TBFI model and 0.68 to 0.82 for the FI model (Figure 1).

### 3.2 Overall survival

During follow‐up, 11% (*n* = 21/189) and 50% (*n* = 94/189) of the patients died, due to any cause, within 30 days and 6 months of the frailty assessment, respectively. When examining frailty independently using FI or TBFI, we found that the probability of survival decreased with higher levels of frailty. The ability to predict mortality was comparable between the two indices. The hazard ratio (HR) was 4.5‐fold (95% CI, 2.49–8.14) and 4.1‐fold (95% CI, 1.69–9.94) higher for mortality among severely frail patients compared to non‐frail patients based on the FI and TBFI, respectively. Further, the HR was 2.2‐fold (95% CI, 1.204, 4.119) and 3.0‐fold (95% CI, 1.290, 7.439) higher for mortality (i.e., poor outcomes) among moderately frail patients compared to non‐frail patients based on the FI and TBFI, respectively.

Age and type of primary cancer were not significantly associated with survival, whereas male sex was a risk factor (Table 2). The association of frailty with long‐term survival (defined as being alive at 6 months from the date of discharge from the index admission) was stronger than short‐term survival (alive at 30 days) (Table S6). The likelihood of death at 6 months was about 11‐fold (odds ratio, 10.9 [95% CI, 3.97–33.24]) and 9.73‐fold (95% CI, 2.85–38.50) higher for severely frail patients compared to non‐frail patients for FI and TBFI, respectively (Table 3).

**TABLE 2:** Multivariable Cox Proportional Hazards Regression Models for OS (FI and TBFI)

Variable	FI Overall survival		TBFI Overall survival	
	HR (95% CI)	*p* Value	HR (95% CI)	*p* Value
Age				
<62 years	1.0 (Reference)	—	1.0 (Reference)	—
≥ 62 years	0.806 (0.543, 1.197)	0.2852	0.905 (0.608, 1.348)	0.6235
Sex				
Female	1.0 (Reference)	—	1.0 (Reference)	—
Male	1.646 (1.073, 2.526)	0.0226	1.608 (1.054, 2.453)	0.0276
Group FI			Group TBFI	
Non‐frail	1.0 (Reference)	—	1.0 (Reference)	—
Mild	1.624 (0.941, 2.804)	0.0817	1.928 (0.777, 4.784)	0.1565
Moderate	2.227 (1.204, 4.119)	0.0107	3.098 (1.290, 7.439)	0.0114
Severe	4.510 (2.519, 8.075)	<0.0001	4.102 (1.692, 9.942)	0.0018
Type of primary cancer				
Breast	1.0 (Reference)	—	1.0 (Reference)	—
GI	1.976 (0.875, 4.463)	0.1012	1.842 (0.803, 4.225)	0.1496
GU	1.240 (0.476, 3.230)	0.6598	1.340 (0.507, 3.541)	0.5548
H&N	1.525 (0.466, 4.987)	0.4855	1.550 (0.476, 5.050)	0.4674
Lung	1.880 (0.826, 4.276)	0.1323	2.096 (0.920, 4.777)	0.0782
Melanoma	2.734 (0.957, 7.817)	0.0605	2.755 (0.965, 7.868)	0.0584
Other	1.307 (0.519, 3.291)	0.5695	1.775 (0.711, 4.431)	0.2186

Abbreviations: FI, frailty index; GI, gastrointestinal; GU, genitourinary; H&N, head and neck; HR, hazard ratio; OS, overall survival; TBFI, test based frailty index.

**TABLE 3:** FI and TBFI 6‐month mortality rates by frailty level

Variable	FI: 6‐month mortality		TBFI: 6‐month mortality	
	OR (95% CI)	*p* Value	OR (95% CI)	*p* Value
Age				
<62 years	1.0 (Reference)	—	1.0 (Reference)	—
≥62 years	0.417 (0.206, 0.818)	0.0126	0.534 (0.269, 1.041)	0.0689
Sex				
Female	1.0 (Reference)	—	1.0 (Reference)	—
Male	2.168 (1.058, 4.550)	0.0370	1.929 (0.956, 3.979)	0.0699
Type of primary cancer				
Breast	1.0 (Reference)	—	1.0 (Reference)	—
GI	2.420 (0.753, 8.421)	0.1480	2.381 (0.744, 8.188)	0.1528
GU	1.204 (0.284, 5.230)	0.8012	2.114 (0.538, 8.696)	0.2885
H&N	1.609 (0.284, 9.265)	0.5877	2.485 (0.464, 14.041)	0.2891
Lung	2.174 (0.636, 7.886)	0.2232	3.335 (1.035, 11.592)	0.0489
Melanoma	3.241 (0.579, 19.848)	0.1865	3.683 (0.654, 22.741)	0.1450
Other	1.461 (0.365, 5.993)	0.5926	2.301 (0.613, 9.097)	0.2223
Group FI				
Non‐frail	1.0 (Reference)	—	1.0 (Reference)	—
Mild	2.011 (0.884, 4.680)	0.0989	2.594 (0.793, 9.709)	0.1307
Moderate	5.506 (2.066, 15.469)	0.0008	4.610 (1.440, 17.040)	0.0140
Severe	10.967 (3.974, 33.236)	<0.0001	9.725 (2.853, 38.501)	0.0005

Multivariable Logistic Regression Models for 6‐month mortality.

Abbreviations: FI, frailty index; GI, gastrointestinal; GU, genitourinary; H&N, head and neck; HR, hazard ratio; OS, overall survival.

**FIGURE 2:**
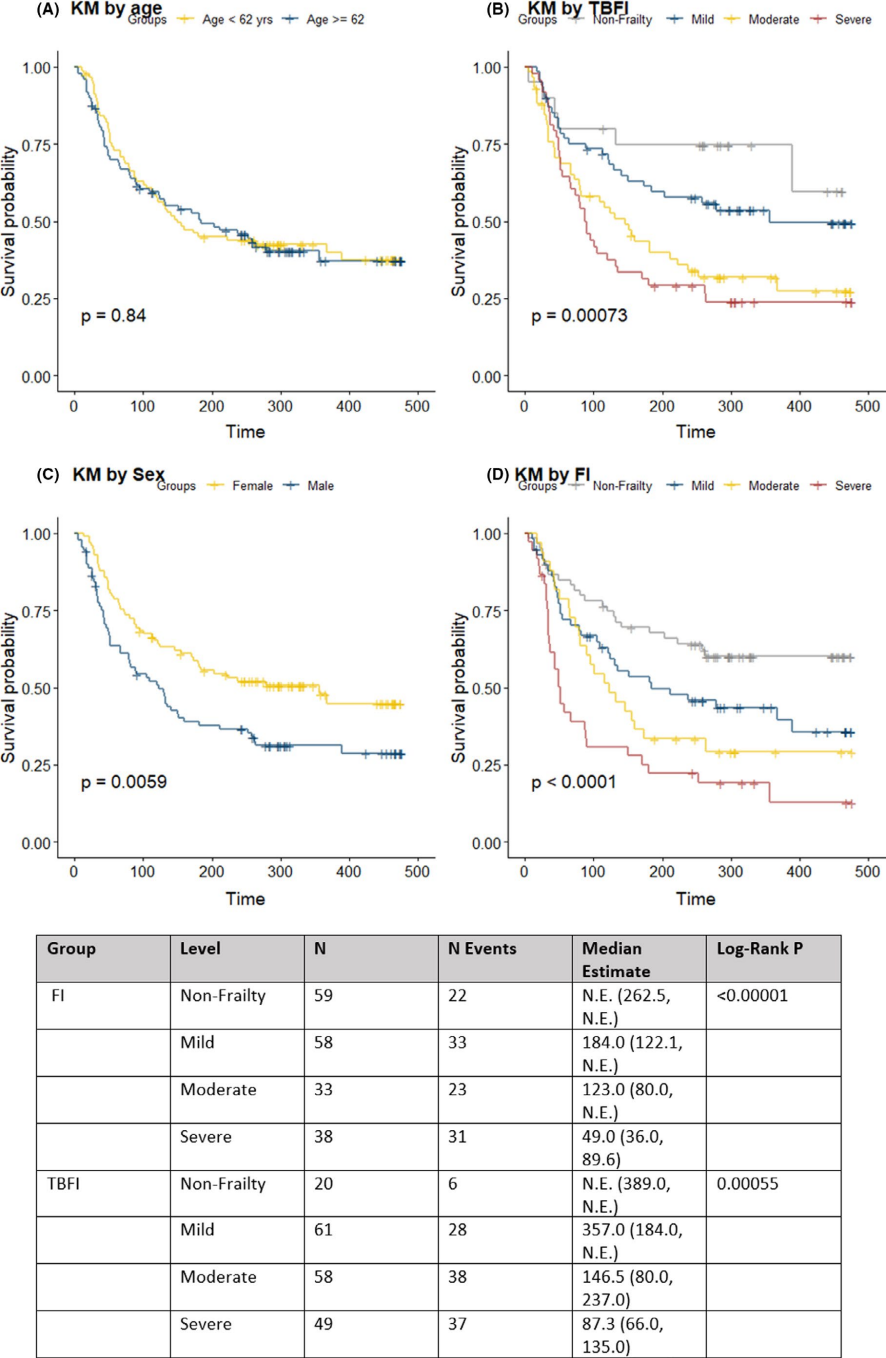
Kaplan–Meier survival curves by age (Panel A), TBFI (Panel B), sex (Panel C), and FI (Panel D)

Figure 2 indicates a trend for better cumulative survival in the non‐frail and mildly frail groups compared with the moderately and severely frail groups. Kaplan–Meier analysis showed that age did not discriminate survival probability, whereas frailty (as indicated by FI or TBFI) and sex did. The median OS for the severely frail group was 49 days (95% CI, 36.0–89.6) and 87 days (95% CI, 66.0–135.0) for FI and TBFI, respectively, with overlapping confidence intervals. The FI was predictive of mortality for older patients (≥62 years) as well as younger patients (<62 years of age) (Figure S4), whereas the TBFI, when stratified by age, was significant only in the younger patient group (Figure S5).

## 4 DISCUSSION

We created a 20 item TBFI from laboratory tests representative of known prognostic indicators in advanced cancer patients (e.g., hypercalcemia, anemia, hyponatremia, and lymphopenia) representing multiple different bodily/organ systems. Our TBFI included cancer‐specific factors, such as liver and brain metastasis. Other lab‐based FIs combining routine blood tests and standard physical measurements, such as blood pressure and pulse, have been developed,^36^, ^37^ but none have been developed that use clinically sensible measures for cancer patients. Previously, Blodgett et al compared a lab‐based FI, a clinical FI (based on the domains of a CGA), and a combination of both and reported that the lab‐based FI predicted adverse health outcomes in a large population of community‐dwelling men^38^; additionally, they suggested that the lab‐based FI may be feasible as a screening tool in the hospital care setting.

Our study was the first to demonstrate that a cancer‐specific TBFI was comparable to a more comprehensive combined clinical‐ and lab‐based FI in predicting survival in the cancer hospital setting. The FI, which had 53 items and included in‐person assessment of elements of the CGA, was laborious and not practical for patient evaluation in the inpatient setting. The TBFI, however, illustrated that a lab‐based FI could be a robust predictor of survival, with items that are focused primarily on objective measures for ease of frailty score calculation and data collection. This would theoretically allow for the electronic medical record to automate a frailty score for patients as they are admitted to the hospital.

Uncomplicated frailty instruments are desirable for use in a busy clinical setting, such as during acute hospitalization, to provide clinical decision support. A tool such as the TBFI would provide a screening assessment of frailty and add to the clinical information known about a hospitalized cancer patient. This type of information will allow for better interdisciplinary communication and goal‐concordant care planning. Constructing a TBFI from laboratory data in the hospital setting may be easier than constructing a FI based on clinical assessment, but it doesn't negate the need for further comprehensive assessment. Screening for moderately frail patients should prompt a search for modifiable risk factors and a referral for further assessment. Identification and correction of a modifiable clinical deficit, thereby returning the patient to a more robust state, may have real implications on cancer outcomes. For severely frail patients, who have anticipated poor outcomes and limited survival, timely goals of care conversations, referral to hospice, and a focus on QOL will improve EOL care and reduce unnecessary hospitalizations.

We found in our model that the association of frailty with 6‐month survival was much stronger than 30‐day survival. We hypothesize that this may be because the severity/acuity of admitting diagnosis was not fully comprehended in this model, and further work needs to be done to understand this finding.

### 4.1 Strengths of this study

A pragmatic decision was made to include patients across a wide range of ages (adults ≥18 years) from the inpatient ward to reflect the reality of our practice as oncological hospitalists.

### 4.2 Limitations of our study

Our study has some limitations. This was a modest single‐center study (*n *= 189), 86.8% of patients were white, and minorities were not well represented, which may limit the generalizability of the results in ways that are unknown to us.

There are many methodological challenges for conducting hospital‐based research of patients with cancer. High levels of fatigue, emotional distress, and pain can influence a patient's ability to participate in a structured interview process. The interviewer‐administered surveys were meant to probe levels of functioning regarding multiple domains (cognitive, physical, social, and emotional). Survey estimates of normative behavior—like church attendance or volunteerism—often include substantial measurement error, as respondents may report higher rates of these behaviors than is warranted. Likewise, rates of counternormative behaviors—like “I need help toileting and cleaning myself”—are underreported.^39^ Additionally, our study included younger cancer patients, which may have biased the responses to overestimate functional capabilities. In general, Theou et al reported that frailty levels are lower when self‐reported items are used.^40^

Acute hospitalization often represents a sudden decrease in a patient's overall level of functioning or worsening symptom control. Interview/survey questions were geared to assess current levels of functioning. If an acute event prompted hospitalization, then the assessment may have overestimated deficits assigned to the individual, as the patient would recover from the acute event and regain functionality. Whereas the calculation of an FI is a metric that should represent the current stable state of the individual, it is likely that our FI captured a combination of stable deficits and deficits associated with acute hospitalization. Likewise, overly pessimistic or optimistic views of the current situation by patients or family members would bias the responses.

## 5 CONCLUSIONS

In conclusion, a TBFI derived from an electronic health record may be practical and useful for predicting survival of hospitalized patients with cancer. The TBFI can be used to both identify vulnerable patients at risk for decline in status (mildly to moderately frail) and patients who are truly EOL (severely frail) among both older and younger patients. The addition of an in‐person assessment using 33 items from the standard CGA did show an improvement in the model's survival prediction, but the magnitude of the improvement was negligible. The TBFI developed in our study showed valuable discriminatory accuracy, as indicated by an AUC >0.70 for 6‐month mortality.

## Conflict of interest

The authors declare no conflicts of interest.

## ETHICAL APPROVAL STATEMENT

Individuals whose data are included in this study database have provided written informed consent to allow their data to be recorded and used for research purposes. The study protocol and consent form were approved by the Institutional Review Board of the University of South Florida (Pro00035916). All participants provided informed written consent prior to enrollment in the study.

## Supporting information

Table S1‐S6‐Fig S1‐S5

## Data Availability

The data that support the findings of this study are available from the corresponding author upon reasonable request.
