# Supplementary material for: A simple test‐based frailty index to predict survival among cancer patients with an unplanned hospitalization: An observational cohort study
Source: Cancer Med. 2021 Aug 5;10(17):5765–74. doi: 10.1002/cam4.4107 (PMC8419777; doi:10.1002/cam4.4107)
Supplement: Supplementary file 1 — Table S1‐S6‐Fig S1‐S5 [file CAM4-10-5765-s001.docx]

**Supplemental Tables and Figures**

1. Supplemental table T1: Test-based frailty index (TBFI)
2. Supplemental table T2: Self-reported frailty index (SRFI)
3. Supplemental table T3: TBFI descriptive statistics by frailty level
4. Supplemental table T4: FI descriptive statistics by frailty level
5. Supplemental table T5: Characteristics of cohort, TBFI and FI
6. Supplemental table T6: FI Index: Thirty-day mortality rates by frailty level
7. Supplemental figure F1: Patient enrollment process
8. Supplemental figure F2: FI distribution and association with age
9. Supplemental figure F3: TBFI distribution and association with age
10. Supplemental figure F4. KM curve for OS by TBFI group for each age group. Panel A: ≥ 62 years of age; Panel B: < 62 years of age
11. Supplemental Figure F5. KM curve for OS by FI group for each age group. Panel A: ≥ 62 years of age; Panel B: < 62 years of age

| Supplemental Table T1: Test Based Frailty Index (TBFI) | | |
| --- | --- | --- |
| Domain | **Item** | **Coding** |
| Healthcare use | No. healthcare visits in last 6 months (not including index admission) | ≥ 2 visits = 1; <2 visits = 0 |
|  | Length of index admission | ≥ 5 day = 1; < 5 day = 0 |
| Test-based factors† | Corrected calcium, mg/dl | ≥ 9.5 = 1; < 9.5 = 0 |
|  | Creatinine, mg/dl | ≥ 1.74 = 1; <1.74 = 0 |
|  | Hemoglobin, g/dl | ≤ 8 = 1; > 8 = 0 |
|  | Sodium, mmol/L | ≤ 132 = 1; > 132 = 0 |
|  | White blood cells, k/µl | ≤ 4 or ≥ 17.5 = 1; (>4, <17.5) = 0 |
|  | Neutrophils, k/µl | ≤ 0.87 = 1; >0.87 = 0 |
|  | Lymphocytes, k/µl | ≤ 1.1 = 1; > 1.1 =0 |
|  | Platelets, k/µl | ≤ 95 or ≥ 350 = 1; (>95, <350) = 0 |
|  | Albumin, g/dl | ≤ 2.5 = 1; >2.5 =0 |
| Inflammatory indices | NLR | ≥ 5 =1; <5 =0 |
|  | PLR | ≥ 250 =1; < 250 = 0 |
|  | SII | ≥ 1600 = 1; < 1600= 0 |
| Malnutrition index | PNI | ≤ 4.5 = 1; >4.5 = 0 |
| Cancer-specific factors | Known diagnosis of metastasis | Yes = 1; No= 0 |
|  | Liver metastasis | Yes = 1; No= 0 |
|  | Brain metastasis | Yes = 1; No= 0 |
| Other | No. of medications | ≥ 5 = 1; < 5 = 0 |
|  | BMI | ≤ 19 or ≥ 35 = 1; (>19, <35) =0 |
| † coding targets (±) 1 SD from the mean (1000 unidentified patient’s data sampled between September 2018 through May 2019) to represent general medical inpatient population values).  Abbreviations: BMI, basal metabolic index; NLR, Neutrophil to Lymphocyte ratio; PLR, platelet to lymphocyte ratio; PNI, prognostic nutrition index; SII, systemic immune-inflammation index; TBFI, test based frailty index. | | |

| Supplemental Table T2: Self-Reported Frailty Index (SFRI) | | |
| --- | --- | --- |
| Domain | **Item** | **Coding** |
| Activities of daily living/ mobility | Bed/ chair bound | Yes = 1 |
|  | Able to get out of bed but do not go out | Yes = 0.5 |
|  | Not bed/ chair bound at all | Yes = 0 |
| Bathing | Able to bathe myself completely or help with only 1 body part | Yes = 0 |
|  | Need help with more than one body part | Yes = 1 |
| Transferring | Able to move in/out of bed/chair unassisted OR with mechanical transfer aids | Yes = 0 |
|  | I need help moving from bed to chair OR require a complete transfer | Yes = 1 |
| Toileting | Able to use toilet, transfer, arrange clothing, clean genital area | Yes = 0 |
|  | Need help transferring, toileting, cleaning OR use bedpan | Yes = 1 |
| Bowel/bladder control | I have complete self-control | Yes = 0 |
|  | I have partial or complete lack of control over my bladder OR bowels | Yes = 1 |
| Feeding | Able to feed myself without help (disregard food preparation) | Yes = 0 |
|  | Partially or completely unable to feed myself OR I require parenteral feeding | Yes = 1 |
| Walking | I use crutches/cane/walker to walk | Yes = 1 |
|  | I do not use crutches/cane/walker but lean on furniture for help when walking | Yes = 0.5 |
|  | I can walk on my own, with no crutches/cane/walker or furniture | Yes = 0 |
|  | When I walk, I use short steps or shuffle, can’t lift my head while walking, OR have a hard time getting up from a chair | Yes = 1 |
|  | I take weak, short steps but can lift my head when walking OR use some furniture support or light touch to guide me | Yes = 0.5 |
|  | I walk unimpaired | Yes = 0 |
| Self-awareness | When I cannot walk on my own, I ask for help | Yes = 0 |
|  | I do not ask for help with walking even when I need it | Yes = 1 |
|  | When it comes to accomplishing physical tasks, sometimes I try to do more than I can | Yes = 1 |
|  | I do not try to do more than I can | Yes = 0 |
| Memory | I have trouble remembering things that happened recently | Yes = 1 |
|  | I do not have trouble remembering things that happened recently | Yes = 0 |
|  | I have trouble recalling conversations a few days later | Yes = 1 |
|  | I do not have trouble recalling conversations a few days later | Yes = 0 |
| ECOG | I am fully active | Yes = 0 |
|  | I am restricted in physically strenuous activity | Yes = 0.25 |
|  | I can move around but only enough to take care of myself and do not do work activities | Yes = 0.5 |
|  | I am only able to do limited self-care OR I am confined to a bed/chair more than 50% of the time | Yes = 0.75 |
|  | I am not able to take care of myself OR I am totally confined to a bed or chair | Yes = 1 |
| Instrumental activities of daily living | I shop on my own | Yes = 0; No = 1 |
|  | I cook for myself | Yes = 0; No = 1 |
|  | I manage my own medications | Yes = 0; No = 1 |
|  | I use the phone on my own | Yes = 0; No = 1 |
|  | I can do housework | Yes = 0; No = 1 |
|  | I can do laundry on my own | Yes = 0; No = 1 |
|  | I can drive or use public transportation | Yes = 0; No = 1 |
|  | I can manage my own finances | Yes = 0; No = 1 |
| Comorbidities | I have severe liver disfunction (ascites, cirrhosis, ESLD) | Yes = 1; No = 0 |
|  | I have moderate to severe renal dysfunction | Yes = 1; No = 0 |
|  | I am hemiplegic | Yes = 1; No = 0 |
|  | I have diabetes | Yes = 1; No = 0 |
|  | I have a history of cardiac problems (heart attack, CHF) | Yes = 1; No = 0 |
|  | I have cognitive impairment (mild dementia) | Yes = 1; No = 0 |
|  | I have COPD | Yes = 1; No = 0 |
| Uncontrolled symptoms | I have uncontrolled pain | Yes = 1; No = 0 |
|  | I have persistent nausea | Yes = 1; No = 0 |
|  | I have depression and anxiety | Yes = 1; No = 0 |
|  | I have significant fatigue | Yes = 1; No = 0 |
|  | I have a poor appetite and poor oral intake of food | Yes = 1; No = 0 |
| Abbreviations: CHF, congestive heart failure; COPD, Chronic obstructive pulmonary disease; ECOG, Eastern Cooperative Oncology Group; ESLD, End-stage Liver Disease. | | |

| **Supplemental Table T3: TBFI descriptive statistics by frailty level.** | | | | | |
| --- | --- | --- | --- | --- | --- |
| **Characteristic** | **Nonfrail,**  **N = 20** | **Mildly frail, N = 62** | **Moderately frail, N = 58** | **Severely frail, N = 49** | **p.overall** |
| **Age [range]** | 59.0 [37.0;87.0] | 64.0 [40.0;84.0] | 65.0 [38.0;87.0] | 58.0 [26.0;79.0] | 0.084 |
| **Age, no. (%)** |  |  |  |  | 0.040 |
| < 62 yrs | 12 (60.0) | 27 (43.5) | 21 (36.2) | 30 (61.2) |  |
| ≥ 62 yrs | 8 (40.0) | 35 (56.5) | 37 (63.8) | 19 (38.8) |  |
| **Sex, no. (%)** |  |  |  |  | 0.547 |
| Female | 10 (50.0) | 36 (58.1) | 26 (44.8) | 25 (51.0) |  |
| Male | 10 (50.0) | 26 (41.9) | 32 (55.2) | 24 (49.0) |  |
| **Type of primary cancer, no. (%)** |  |  |  |  | 0.069 |
| Breast | 2 (10.0) | 13 (21.0) | 3 (5.17) | 7 (14.3) |  |
| Gastrointestinal | 9 (45.0) | 12 (19.4) | 19 (32.8) | 14 (28.6) |  |
| Genitourinary | 1 (5.00) | 10 (16.1) | 9 (15.5) | 7 (14.3) |  |
| Head and neck | 2 (10.0) | 2 (3.23) | 4 (6.90) | 2 (4.08) |  |
| Lung | 3 (15.0) | 13 (21.0) | 9 (15.5) | 14 (28.6) |  |
| Melanoma | 1 (5.00) | 4 (6.45) | 4 (6.90) | 2 (4.08) |  |
| Other | 2 (10.0) | 8 (12.9) | 10 (17.2) | 3 (6.12) |  |
| **Palliative care in prior 6 months, no. (%)** |  |  |  |  | 0.059 |
| No | 18 (90.0) | 56 (90.3) | 42 (72.4) | 39 (79.6) |  |
| Yes | 2 (10.0) | 6 (9.68) | 16 (27.6) | 10 (20.4) |  |
| **Palliative care consult during index admission, no. (%)** |  |  |  |  |  |
| Symptom management |  |  |  |  | 0.199 |
| No | 12 (60.0) | 51 (82.3) | 41 (70.7) | 37 (75.5) |  |
| Yes | 8 (40.0) | 11 (17.7) | 17 (29.3) | 12 (24.5) |  |
| Goals of care |  |  |  |  | 0.065 |
| No | 20 (100) | 62 (100) | 54 (93.1) | 45 (91.8) |  |
| Yes | 0 (0.00) | 0 (0.00) | 4 (6.90) | 4 (8.16) |  |
| **Healthcare visits in the prior 6 months, no. [range]** | 1.00 [0.00;5.00] | 1.00 [0.00;12.0] | 1.50 [0.00;12.0] | 3.00 [0.00;20.0] | <0.001 |
| **Healthcare visits deficit category (≥ 2), no. (%)** |  |  |  |  | <0.001 |
| 0 | 16 (80.0) | 37 (59.7) | 29 (50.0) | 13 (26.5) |  |
| 1 | 4 (20.0) | 25 (40.3) | 29 (50.0) | 36 (73.5) |  |
| **Prior admission within 30 days, no. (%)** |  |  |  |  | <0.001 |
| No | 17 (85.0) | 52 (83.9) | 38 (65.5) | 23 (46.9) |  |
| Yes | 3 (15.0) | 10 (16.1) | 20 (34.5) | 26 (53.1) |  |
| **Distance from MCC in miles**  **Average [range]** | 35.0 [7.50;250] | 31.4 [2.00;500] | 34.3 [5.00;1250] | 37.7 [4.00;1300] | 0.783 |
| **Length of index admission** | 3.50 [1.00;7.00] | 5.00 [1.00;28.0] | 6.50 [2.00;28.0] | 6.00 [1.00;17.0] | <0.001 |
| **Presences or absence of deficit, no. (%)** |  |  |  |  |  |
| Length of Stay deficit (≥5) |  |  |  |  | <0.001 |
| 0 | 14 (70.0) | 29 (46.8) | 15 (25.9) | 12 (24.5) |  |
| 1 | 6 (30.0) | 33 (53.2) | 43 (74.1) | 37 (75.5) |  |
| Corrected Calcium DV (≥9.5) |  |  |  |  | <0.001 |
| 0 | 17 (85.0) | 34 (54.8) | 24 (41.4) | 12 (24.5) |  |
| 1 | 3 (15.0) | 28 (45.2) | 34 (58.6) | 37 (75.5) |  |
| Creatinine DV (>1.74) |  |  |  |  | 0.753 |
| 0 | 18 (90.0) | 57 (91.9) | 52 (89.7) | 42 (85.7) |  |
| 1 | 2 (10.0) | 5 (8.06) | 6 (10.3) | 7 (14.3) |  |
| Medications DV (≥5) |  |  |  |  | <0.001 |
| 0 | 10 (50.0) | 8 (12.9) | 12 (20.7) | 0 (0.00) |  |
| 1 | 10 (50.0) | 54 (87.1) | 46 (79.3) | 49 (100) |  |
| BMI DV (<19 or >35) |  |  |  |  | 0.218 |
| 0 | 18 (90.0) | 53 (85.5) | 46 (79.3) | 35 (71.4) |  |
| 1 | 2 (10.0) | 9 (14.5) | 12 (20.7) | 14 (28.6) |  |
| Brain Metastasis DV (Y=1) |  |  |  |  | 0.011 |
| 0 | 20 (100) | 52 (83.9) | 52 (89.7) | 35 (71.4) |  |
| 1 | 0 (0.00) | 10 (16.1) | 6 (10.3) | 14 (28.6) |  |
| Liver Metastasis DV (Y=1) |  |  |  |  | <0.001 |
| 0 | 19 (95.0) | 57 (91.9) | 41 (70.7) | 27 (55.1) |  |
| 1 | 1 (5.00) | 5 (8.06) | 17 (29.3) | 22 (44.9) |  |
| Serum Sodium DV (<132) |  |  |  |  | 0.153 |
| 0 | 19 (95.0) | 55 (88.7) | 50 (86.2) | 37 (75.5) |  |
| 1 | 1 (5.00) | 7 (11.3) | 8 (13.8) | 12 (24.5) |  |
| WBC DV (>17.5 or <4) |  |  |  |  | 0.010 |
| 0 | 19 (95.0) | 47 (75.8) | 48 (82.8) | 30 (61.2) |  |
| 1 | 1 (5.00) | 15 (24.2) | 10 (17.2) | 19 (38.8) |  |
| Neutrophil count DV (<0.87) |  |  |  |  | 0.958 |
| 0 | 20 (100) | 59 (95.2) | 55 (94.8) | 47 (95.9) |  |
| 1 | 0 (0.00) | 3 (4.84) | 3 (5.17) | 2 (4.08) |  |
| Lymphocyte count DV (<1.1) |  |  |  |  | <0.001 |
| 0 | 17 (85.0) | 29 (46.8) | 11 (19.0) | 3 (6.12) |  |
| 1 | 3 (15.0) | 33 (53.2) | 47 (81.0) | 46 (93.9) |  |
| Platelet count DV (<95 or >350) |  |  |  |  | <0.001 |
| 0 | 13 (65.0) | 52 (83.9) | 35 (60.3) | 16 (32.7) |  |
| 1 | 7 (35.0) | 10 (16.1) | 23 (39.7) | 33 (67.3) |  |
| Albumin DV (< 2.5) |  |  |  |  | 0.065 |
| 0 | 20 (100) | 60 (96.8) | 56 (96.6) | 42 (85.7) |  |
| 1 | 0 (0.00) | 2 (3.23) | 2 (3.45) | 7 (14.3) |  |
| NLR DV (≥5) |  |  |  |  | <0.001 |
| 0 | 13 (65.0) | 30 (48.4) | 14 (24.1) | 5 (10.2) |  |
| 1 | 7 (35.0) | 32 (51.6) | 44 (75.9) | 44 (89.8) |  |
| PLR DV (≥250) |  |  |  |  | <0.001 |
| 0 | 13 (65.0) | 30 (48.4) | 15 (25.9) | 11 (22.4) |  |
| 1 | 7 (35.0) | 32 (51.6) | 43 (74.1) | 38 (77.6) |  |
| PNI DV (≤4.5) |  |  |  |  | <0.001 |
| 0 | 14 (70.0) | 24 (38.7) | 8 (13.8) | 1 (2.04) |  |
| 1 | 6 (30.0) | 38 (61.3) | 50 (86.2) | 48 (98.0) |  |
| SII_DV (≥1600) |  |  |  |  | <0.001 |
| 0 | 12 (60.0) | 35 (56.5) | 20 (34.5) | 9 (18.4) |  |
| 1 | 8 (40.0) | 27 (43.5) | 38 (65.5) | 40 (81.6) |  |
| **Ratio of Social Deficit Values [range]** | 0.40 [0.00;0.53] | 0.27 [0.00;0.67] | 0.27 [0.07;0.67] | 0.27 [0.00;0.80] | 0.292 |
| **Metabolic Cancer-Specific Deficits [range]** | 3.00 [1.00;4.00] | 5.00 [3.00;6.00] | 6.00 [5.00;8.00] | 8.00 [7.00;11.0] | <0.001 |
| **Primary ADLs (questions 38 through 50) [range]** | 2.25 [0.00;11.0] | 1.88 [0.00;10.2] | 2.12 [0.00;9.50] | 3.25 [0.00;9.00] | 0.085 |
| **Secondary ADLs (questions 51 through 58) [range]** | 0.00 [0.00;8.00] | 0.00 [0.00;8.00] | 0.00 [0.00;8.00] | 2.00 [0.00;7.00] | 0.110 |
| **Comorbidities (questions 59 through 65) [range]** | 0.00 [0.00;1.00] | 1.00 [0.00;4.00] | 0.00 [0.00;3.00] | 0.00 [0.00;3.00] | 0.043 |
| **Uncontrolled symptoms (questions 66 through 70) [range]** | 2.00 [0.00;5.00] | 2.00 [0.00;5.00] | 2.00 [0.00;5.00] | 3.00 [0.00;6.00] | 0.006 |
| **Social deficits (questions 71 through 85) [range]** | 6.00 [0.00;8.00] | 4.00 [0.00;10.0] | 4.00 [1.00;10.0] | 4.00 [0.00;12.0] | 0.292 |
| **Race, no. (%)** |  |  |  |  | 0.053 |
| African American/African/Black/Caribbean | 1 (5.00) | 4 (6.45) | 2 (3.45) | 9 (18.4) |  |
| Asian/Pacific Islander | 0 (0.00) | 1 (1.61) | 0 (0.00) | 2 (4.08) |  |
| Native American/American Indian | 0 (0.00) | 0 (0.00) | 1 (1.72) | 0 (0.00) |  |
| Other | 0 (0.00) | 0 (0.00) | 3 (5.17) | 2 (4.08) |  |
| White | 19 (95.0) | 57 (91.9) | 52 (89.7) | 36 (73.5) |  |
| **Presence of metastases, no. (%)** |  |  |  |  | <0.001 |
| 0 | 9 (45.0) | 26 (41.9) | 5 (8.62) | 7 (14.3) |  |
| 1 | 11 (55.0) | 36 (58.1) | 53 (91.4) | 42 (85.7) |  |
| **Readmission time** | 164 [3.00;441] | 52.0 [2.00;476] | 42.0 [4.00;475] | 42.0 [1.00;468] | 0.295 |
| **Abbreviations**: ADL, activity of daily living; BMI, basal metabolic index; DV, deficit value; MCC, Moffitt Cancer Center; NLR, neutrophil-lymphocyte ratio; PLR, platelet-to-lymphocyte ratio; PLT, platelet lymphocyte ratio; PNI, prognostic nutrition index; SII, systemic immune-inflammation index; WBC, white blood cells; 0 indicates the absence of a deficit and 1 indicates the presence of a deficit. | | | | | |

| **Supplemental Table T4: FI descriptive statistics by frailty level** | | | | | |
| --- | --- | --- | --- | --- | --- |
| **Characteristics** | **Nonfrail,**  **N = 59** | **Mildly frail, N = 58** | **Moderately frail, N = 34** | **Severely frail, N = 38** | **p.overall** |
| **Age, y, Avg [range]** | 61.0 [29.0;84.0] | 64.0 [26.0;81.0] | 61.5 [29.0;81.0] | 66.0 [45.0;87.0] | 0.087 |
| **Age, no. (%)** |  |  |  |  | 0.214 |
| < 62 years | 33 (55.9) | 27 (46.6) | 17 (50.0) | 13 (34.2) |  |
| ≥ 62 years | 26 (44.1) | 31 (53.4) | 17 (50.0) | 25 (65.8) |  |
| **Sex, no. (%)** |  |  |  |  | 0.604 |
| Female | 34 (57.6) | 28 (48.3) | 18 (52.9) | 17 (44.7) |  |
| Male | 25 (42.4) | 30 (51.7) | 16 (47.1) | 21 (55.3) |  |
| **Type of primary cancer, no. (%)** |  |  |  |  | 0.072 |
| Breast | 13 (22.0) | 6 (10.3) | 3 (8.82) | 3 (7.89) |  |
| Gastrointestinal | 20 (33.9) | 22 (37.9) | 6 (17.6) | 6 (15.8) |  |
| Genitourinary | 5 (8.47) | 6 (10.3) | 6 (17.6) | 10 (26.3) |  |
| Head and neck | 3 (5.08) | 3 (5.17) | 3 (8.82) | 1 (2.63) |  |
| Lung | 7 (11.9) | 11 (19.0) | 12 (35.3) | 9 (23.7) |  |
| Melanoma | 4 (6.78) | 4 (6.90) | 0 (0.00) | 3 (7.89) |  |
| Other | 7 (11.9) | 6 (10.3) | 4 (11.8) | 6 (15.8) |  |
| **Palliative care in prior 6 months, no. (%)** |  |  |  |  | 0.044 |
| No | 53 (89.8) | 46 (79.3) | 23 (67.6) | 33 (86.8) |  |
| Yes | 6 (10.2) | 12 (20.7) | 11 (32.4) | 5 (13.2) |  |
| **Palliative care during index admission, no. (%)** |  |  |  |  | 0.756 |
| Symptom management |  |  |  |  |  |
| No | 46 (78.0) | 44 (75.9) | 25 (73.5) | 26 (68.4) |  |
| Yes | 13 (22.0) | 14 (24.1) | 9 (26.5) | 12 (31.6) |  |
| Goals of Care |  |  |  |  | 0.017 |
| No | 59 (100) | 56 (96.6) | 33 (97.1) | 33 (86.8) |  |
| Yes | 0 (0.00) | 2 (3.45) | 1 (2.94) | 5 (13.2) |  |
| **Number of health care visits in the prior 6 months** | 1.00 [0.00;12.0] | 2.00 [0.00;10.0] | 2.00 [0.00;7.00] | 3.00 [0.00;20.0] | <0.001 |
| **Health care visits deficit category (≥2), no. (%)** |  |  |  |  | 0.006 |
| 0 | 40 (67.8) | 28 (48.3) | 14 (41.2) | 13 (34.2) |  |
| 1 | 19 (32.2) | 30 (51.7) | 20 (58.8) | 25 (65.8) |  |
| **Prior admission within 30 days, no. (%)** |  |  |  |  | 0.090 |
| No | 48 (81.4) | 37 (63.8) | 22 (64.7) | 23 (60.5) |  |
| Yes | 11 (18.6) | 21 (36.2) | 12 (35.3) | 15 (39.5) |  |
| **Distance from MCC** | 35.0 [5.00;1250] | 32.6 [5.00;1300] | 38.5 [4.00;800] | 32.5 [2.00;1000] | 0.973 |
| **Length of index admission** | 4.00 [1.00;11.0] | 5.00 [1.00;28.0] | 6.00 [2.00;28.0] | 7.50 [2.00;21.0] | <0.001 |
| **Presences or absence of deficit, no. (%)** |  |  |  |  |  |
| Length of Stay deficit (≥5) |  |  |  |  | <0.001 |
| 0 | 31 (52.5%) | 25 (43.1%) | 8 (23.5%) | 6 (15.8%) |  |
| 1 | 28 (47.5%) | 33 (56.9%) | 26 (76.5%) | 32 (84.2%) |  |
| Corrected Calcium DV (≥9.5) |  |  |  |  | <0.001 |
| 0 | 39 (66.1%) | 31 (53.4%) | 13 (38.2%) | 4 (10.5%) |  |
| 1 | 20 (33.9%) | 27 (46.6%) | 21 (61.8%) | 34 (89.5%) |  |
| Creatinine DV (>1.74) |  |  |  |  | 0.512 |
| 0 | 54 (91.5%) | 53 (91.4%) | 28 (82.4%) | 34 (89.5%) |  |
| 1 | 5 (8.47%) | 5 (8.62%) | 6 (17.6%) | 4 (10.5%) |  |
| Medications DV (≥5) |  |  |  |  | 0.787 |
| 0 | 11 (18.6%) | 10 (17.2%) | 4 (11.8%) | 5 (13.2%) |  |
| 1 | 48 (81.4%) | 48 (82.8%) | 30 (88.2%) | 33 (86.8%) |  |
| BMI DV (<19 or >35) |  |  |  |  | 0.869 |
| 0 | 47 (79.7%) | 47 (81.0%) | 26 (76.5%) | 32 (84.2%) |  |
| 1 | 12 (20.3%) | 11 (19.0%) | 8 (23.5%) | 6 (15.8%) |  |
| Brain Metastasis DV (Y=1, N=0) |  |  |  |  | 0.017 |
| 0 | 55 (93.2%) | 51 (87.9%) | 25 (73.5%) | 28 (73.7%) |  |
| 1 | 4 (6.78%) | 7 (12.1%) | 9 (26.5%) | 10 (26.3%) |  |
| Liver Metastasis DV (Y=1, N=0) |  |  |  |  | 0.025 |
| 0 | 53 (89.8%) | 42 (72.4%) | 24 (70.6%) | 25 (65.8%) |  |
| 1 | 6 (10.2%) | 16 (27.6%) | 10 (29.4%) | 13 (34.2%) |  |
| Serum Sodium DV (<132) |  |  |  |  | 0.687 |
| 0 | 52 (88.1%) | 47 (81.0%) | 30 (88.2%) | 32 (84.2%) |  |
| 1 | 7 (11.9%) | 11 (19.0%) | 4 (11.8%) | 6 (15.8%) |  |
| WBC DV (>17.5 or <4) |  |  |  |  | 0.351 |
| 0 | 42 (71.2%) | 49 (84.5%) | 25 (73.5%) | 28 (73.7%) |  |
| 1 | 17 (28.8%) | 9 (15.5%) | 9 (26.5%) | 10 (26.3%) |  |
| Neutrophil count DV (<0.87) |  |  |  |  | 0.155 |
| 0 | 55 (93.2%) | 57 (98.3%) | 31 (91.2%) | 38 (100%) |  |
| 1 | 4 (6.78%) | 1 (1.72%) | 3 (8.82%) | 0 (0.00%) |  |
| Lymphocyte count DV (<1.1) |  |  |  |  | 0.198 |
| 0 | 23 (39.0%) | 18 (31.0%) | 6 (17.6%) | 13 (34.2%) |  |
| 1 | 36 (61.0%) | 40 (69.0%) | 28 (82.4%) | 25 (65.8%) |  |
| Platelet count DV (<95 or >350) |  |  |  |  | 0.196 |
| 0 | 40 (67.8%) | 38 (65.5%) | 20 (58.8%) | 18 (47.4%) |  |
| 1 | 19 (32.2%) | 20 (34.5%) | 14 (41.2%) | 20 (52.6%) |  |
| Albumin DV (< 2.5) |  |  |  |  | 0.347 |
| 0 | 58 (98.3%) | 54 (93.1%) | 31 (91.2%) | 35 (92.1%) |  |
| 1 | 1 (1.69%) | 4 (6.90%) | 3 (8.82%) | 3 (7.89%) |  |
| NLR DV (≥5) |  |  |  |  | 0.076 |
| 0 | 27 (45.8%) | 17 (29.3%) | 8 (23.5%) | 10 (26.3%) |  |
| 1 | 32 (54.2%) | 41 (70.7%) | 26 (76.5%) | 28 (73.7%) |  |
| PLR DV (≥ 250) |  |  |  |  | 0.015 |
| 0 | 31 (52.5%) | 17 (29.3%) | 8 (23.5%) | 13 (34.2%) |  |
| 1 | 28 (47.5%) | 41 (70.7%) | 26 (76.5%) | 25 (65.8%) |  |
| PNI DV (≤4.5) |  |  |  |  | 0.398 |
| 0 | 19 (32.2%) | 14 (24.1%) | 6 (17.6%) | 8 (21.1%) |  |
| 1 | 40 (67.8%) | 44 (75.9%) | 28 (82.4%) | 30 (78.9%) |  |
| SII DV (≥1600) |  |  |  |  | 0.004 |
| 0 | 35 (59.3%) | 19 (32.8%) | 9 (26.5%) | 13 (34.2%) |  |
| 1 | 24 (40.7%) | 39 (67.2%) | 25 (73.5%) | 25 (65.8%) |  |
| **Ratio of social deficit values** | 0.20 [0.00;0.67] | 0.33 [0.00;0.80] | 0.33 [0.07;0.67] | 0.33 [0.00;0.67] | 0.002 |
| **Metabolic cancer-specific deficits** | 5.00 [1.00;9.00] | 6.00 [2.00;9.00] | 7.00 [2.00;10.0] | 7.00 [2.00;11.0] | <0.001 |
| **Primary ADLs (questions 38 through 50)** | 1.00 [0.00;3.00] | 2.25 [0.25;5.50] | 3.62 [0.25;8.00] | 7.75 [3.25;11.0] | <0.001 |
| **Secondary ADLs (questions 51 through 58)** | 0.00 [0.00;3.00] | 0.00 [0.00;5.00] | 2.00 [0.00;8.00] | 6.00 [2.00;8.00] | <0.001 |
| **Comorbidities (questions 59 through 65)** | 0.00 [0.00;2.00] | 0.00 [0.00;3.00] | 1.00 [0.00;4.00] | 1.00 [0.00;3.00] | 0.005 |
| **Uncontrolled symptoms (questions 66 through 70)** | 1.00 [0.00;3.00] | 2.00 [0.00;5.00] | 3.00 [0.00;5.00] | 3.00 [0.00;6.00] | <0.001 |
| **Social deficits (questions 71 through 85)** | 3.00 [0.00;10.0] | 5.00 [0.00;12.0] | 5.00 [1.00;10.0] | 5.00 [0.00;10.0] | 0.002 |
| **Race, no. (%)** |  |  |  |  | 0.672 |
| African American/African/Black/Caribbean | 5 (8.47) | 5 (8.62) | 1 (2.94) | 5 (13.2) |  |
| Asian/Pacific Islander | 1 (1.69) | 1 (1.72) | 1 (2.94) | 0 (0.00) |  |
| Native American/American Indian | 1 (1.69) | 0 (0.00) | 0 (0.00) | 0 (0.00) |  |
| Other | 0 (0.00) | 2 (3.45) | 2 (5.88) | 1 (2.63) |  |
| White | 52 (88.1) | 50 (86.2) | 30 (88.2) | 32 (84.2) |  |
| **Metastasis, no. (%)** |  |  |  |  | 0.007 |
| 0 | 23 (39.0) | 8 (13.8) | 10 (29.4) | 6 (15.8) |  |
| 1 | 36 (61.0) | 50 (86.2) | 24 (70.6) | 32 (84.2) |  |
| **Readmission time** | 72.2 [3.00;475] | 49.0 [2.00;468] | 49.5 [1.00;476] | 34.5 [5.00;468] | 0.623 |
| **Abbreviations**: ADL, activity of daily living; BMI, basal metabolic index; DV, deficit value; MCC, Moffitt Cancer Center; NLR, neutrophil-lymphocyte ratio; PLR, platelet-to-lymphocyte ratio; PLT, platelet lymphocyte ration; PNI, prognostic nutrition index; SII, systemic immune-inflammation index; WBC, white blood cells. | | | | | |

| **Supplemental Table T5: Characteristics of the cohort, TBFI and FI** | | | | | | |
| --- | --- | --- | --- | --- | --- | --- |
| **Characteristics** | **Mean (±SD)** | **No. (%)** | **Median** | **IQR** | **Range** | |
|  |  |  |  |  | **min** | **max** |
| **Age, y** | **61.66 (12.01)** |  | 62 | 55, 71 | 26 | 87 |
| **Sex** |  |  |  |  |  |  |
| Male |  | 92/189 (48.7) |  |  |  |  |
| Female |  | 97/189 (51.3) |  |  |  |  |
| **Type of primary cancer** |  |  |  |  |  |  |
| Breast |  | 25/189(13.2) |  |  |  |  |
| Gastrointestinal |  | 54/189 (28.6) |  |  |  |  |
| Genitorunary |  | 27/189(14.3) |  |  |  |  |
| Head and neck |  | 10/189 (5.3) |  |  |  |  |
| Lung |  | 39/189 (20.6) |  |  |  |  |
| Melanoma |  | 11/189 (5.82) |  |  |  |  |
| Other |  | 23/189 (12.2) |  |  |  |  |
| **TBFI (0-1)** | 0.3505 (0.107) |  | 0.35 | 0.3, 0.45 | 0.05 | 0.6 |
| **TBFI groups** |  |  |  |  |  |  |
| Nonfrail (0-0.2) |  | 20/189 (10.5) |  |  |  |  |
| Mildly frail (0.2-0.3) |  | 62/189 (32.8) |  |  |  |  |
| Moderately frail (0.3-0.4) |  | 58/189 (30.7) |  |  |  |  |
| Severely frail (>0.4) |  | 49/189 (25.9) |  |  |  |  |
| **FI (0-1)** | 0.282 (0.125) |  | 0.264 | 0.18, 0.36 | 0.075 | 0.57 |
| **FI groups** |  |  |  |  |  |  |
| Nonfrail (0-0.2) |  | 59/189 (31.2) |  |  |  |  |
| Mildly frail (0.2-0.3) |  | 58/189 (30.6) |  |  |  |  |
| Moderately frail (0.3-0.4) |  | 34/189 (17.9) |  |  |  |  |
| Severely frail (>0.4) |  | 38/189 (20.1) |  |  |  |  |
| **30-day mortality** |  | 21/189 (11) |  |  |  |  |
| **6-month mortality** |  | 94/189 (50) |  |  |  |  |
| **Abbreviations**: FI, frailty index; IQR, interquartile range; SD, standard deviation; TBFI, test-based frailty index. | | | | | | |

**Supplemental Figure F1**: Patient enrollment process

**Supplemental Figure F2**: FI distribution and association with age


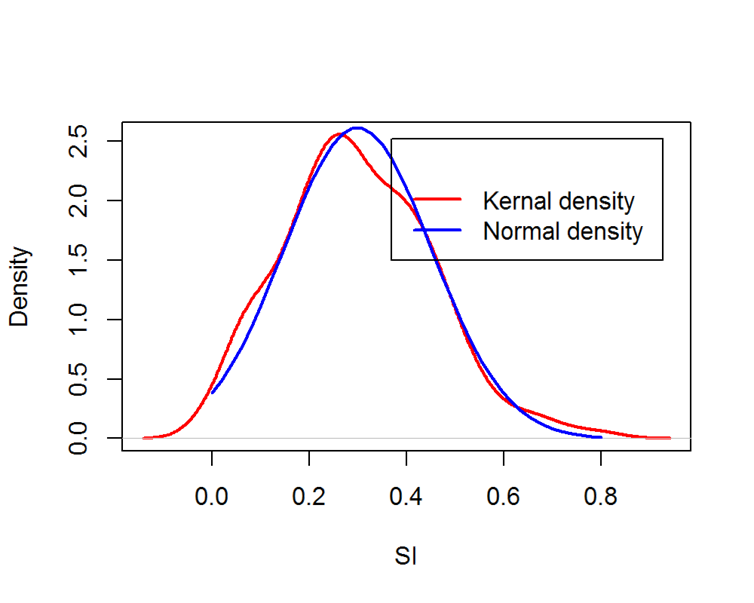


Distribution of **FI**, Shapiro-Wilk’s test, p = 0.0059. Mean 0.282, Median 0.264, SD .125


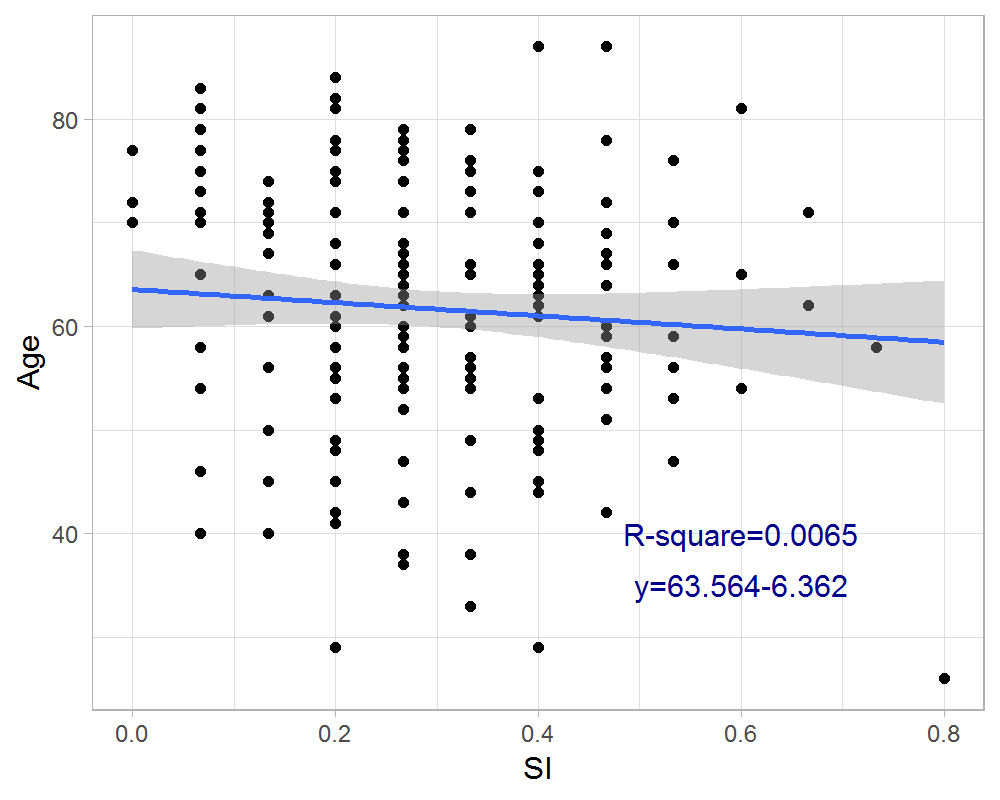


F statistic of the simple linear regression model is 1.231 with p=0.2687

**Supplemental Figure F3:** TBFI distribution and association with age


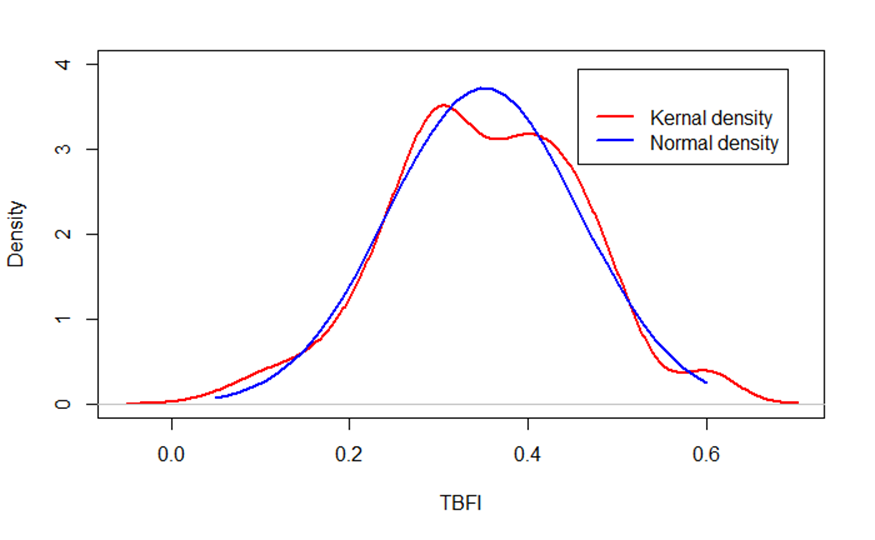


Distribution of **TBFI**; Shapiro-Wilk’s test, p = 0.001, Mean 0.3505, Median 0.35, SD .107


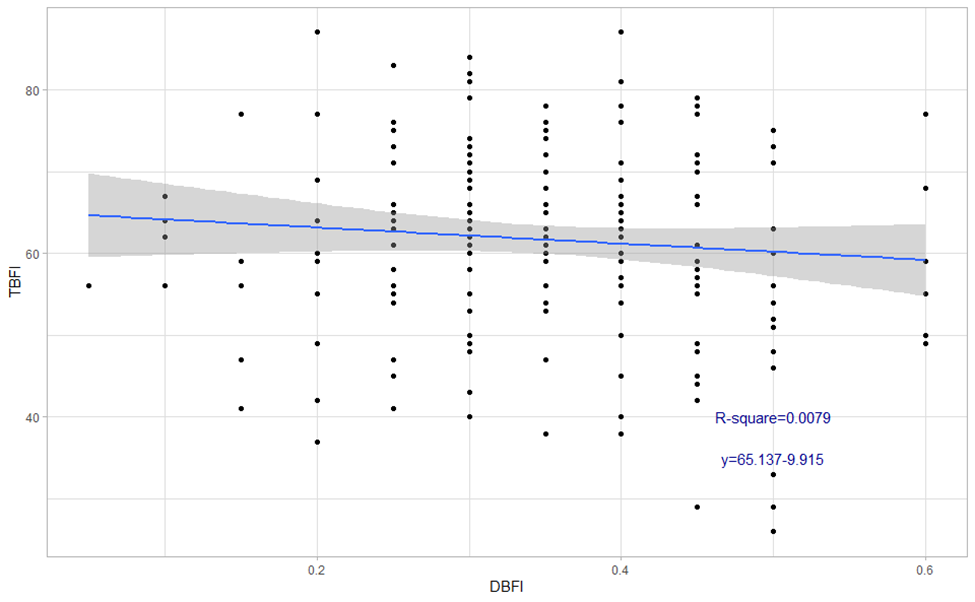


F statistic of the simple linear regression model is 1.48 with p=0. 2253.

**Supplemental Figure F4:** KM curve for OS by FI group for each age group. Panel A: ≥ 62 years of age; Panel B: < 62 years of age


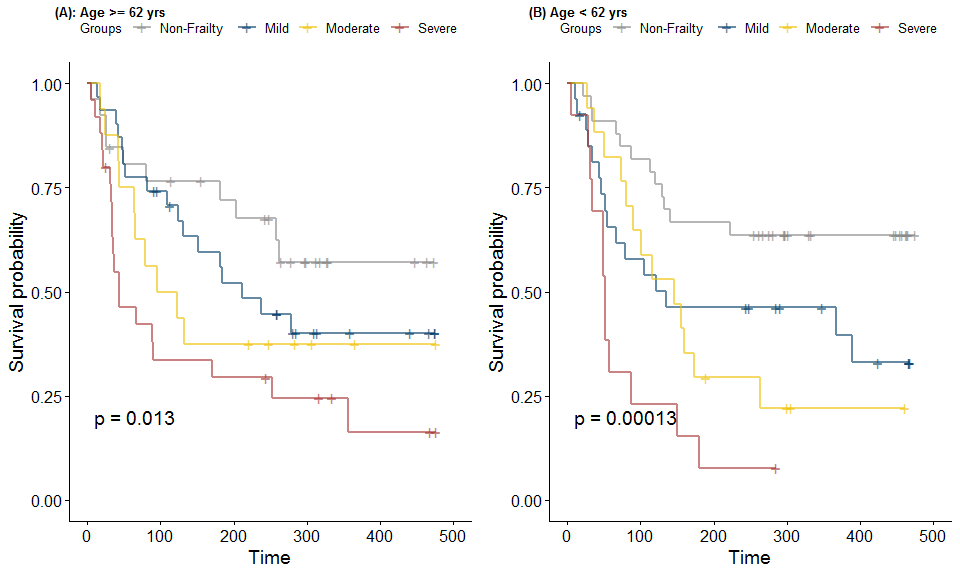


| Group | Level | N | N Events | Median Estimate | Log-Rank P |
| --- | --- | --- | --- | --- | --- |
| >= 62 | Non-Frailty | 26 | 10 | N.E. (258.0, N.E.) | 0.01330 |
|  | Mild | 31 | 17 | 212.0 (131.1, N.E.) |  |
|  | Moderate | 16 | 10 | 109.0 (64.0, N.E.) |  |
|  | Severe | 25 | 19 | 43.0 (34.5, 357.0) |  |
| <= 62 | Non-Frailty | 33 | 12 | N.E. (222.0, N.E.) | 0.00013 |
|  | Mild | 27 | 16 | 135.0 (54.5, N.E.) |  |
|  | Moderate | 17 | 13 | 146.5 (90.0, N.E.) |  |
|  | Severe | 13 | 12 | 52.0 (34.0, N.E.) |  |

**Supplemental Figure F5**
